# Ongoing Outbreak of Extensively Drug-Resistant *Campylobacter jejuni* Infections Associated With US Pet Store Puppies, 2016-2020

**DOI:** 10.1001/jamanetworkopen.2021.25203

**Published:** 2021-09-15

**Authors:** Louise K. Francois Watkins, Mark E. Laughlin, Lavin A. Joseph, Jessica C. Chen, Megin Nichols, Colin Basler, Robert Breazu, Christy Bennett, Lia Koski, Martha P. Montgomery, Michael J. Hughes, Scott Robertson, Charlotte G. Lane, Amber J. Singh, Danielle Stanek, Ellen Salehi, Eric Brandt, Glen McGillivary, Jade Mowery, Jamie DeMent, Rachael D. Aubert, Aimee L. Geissler, Sietske de Fijter, Ian T. Williams, Cindy R. Friedman

**Affiliations:** 1 Division of Foodborne, Waterborne, and Environmental Diseases, Centers for Disease Control and Prevention, Atlanta, Georgia; 2 Now with One Health Office, Centers for Disease Control and Prevention, Atlanta, Georgia; 3 Oak Ridge Institute for Science and Education, Oak Ridge, Tennessee; 4 IHRC Inc, Atlanta, Georgia; 5 CAITTA Inc, Herndon, Virginia; 6 Now with Maricopa County Department of Public Health, Phoenix, Arizona; 7 Epidemic Intelligence Service, Centers for Disease Control and Prevention, Atlanta, Georgia; 8 Ohio Department of Health, Columbus; 9 Now with Division of Viral Hepatitis, Centers for Disease Control and Prevention, Atlanta, Georgia; 10 Florida Department of Health, Tallahassee, Florida; 11 Now with Center for Preparedness and Response, Centers for Disease Control and Prevention, Atlanta, Georgia; 12 Now with Division of Global Migration and Quarantine, Centers for Disease Control and Prevention, Atlanta, Georgia

## Abstract

**Importance:**

Extensively drug-resistant *Campylobacter jejuni* infections cannot be treated with any commonly recommended antibiotics and pose an increasing public health threat.

**Objectives:**

To investigate cases of extensively drug-resistant *C jejuni* associated with pet store puppies and describe the epidemiologic and laboratory characteristics of these infections.

**Design, Setting, and Participants:**

In August 2017, health officials identified, via survey, patients with *C jejuni* infections who reported contact with puppies sold by pet stores. In conjunction with state and federal partners, the Centers for Disease Control and Prevention investigated cases of culture-confirmed *C jejuni* infections in US patients with an epidemiologic or molecular association with pet store puppies between January 1, 2016, and February 29, 2020. Available records from cases occurring before 2016 with genetically related isolates were also obtained.

**Main Outcomes and Measures:**

Patients were interviewed about demographic characteristics, health outcomes, and dog exposure during the 7 days before illness onset. Core genome multilocus sequence typing was used to assess isolate relatedness, and genomes were screened for resistance determinants to predict antibiotic resistance. Isolates resistant to fluoroquinolones, macrolides, and 3 or more additional antibiotic classes were considered to be extensively drug resistant. Cases before 2016 were identified by screening all sequenced isolates submitted for surveillance using core genome multilocus sequence typing.

**Results:**

A total of 168 patients (median [interquartile range] age, 37 [19.5-51.0] years; 105 of 163 female [64%]) with an epidemiologic or molecular association with pet store puppies were studied. A total of 137 cases occurred from January 1, 2016, to February 29, 2020, with 31 additional cases dating back to 2011. Overall, 117 of 121 patients (97%) reported contact with a dog in the week before symptom onset, of whom 69 of 78 (88%) with additional information reported contact with a pet store puppy; 168 isolates (88%) were extensively drug resistant. Traceback investigation did not implicate any particular breeder, transporter, distributer, store, or chain.

**Conclusions and Relevance:**

Strains of extensively drug-resistant *C jejuni* have been circulating since at least 2011 and are associated with illness among pet store customers, employees, and others who come into contact with pet store puppies. The results of this study suggest that practitioners should ask about puppy exposure when treating patients with *Campylobacter* infection, especially when they do not improve with routine antibiotics, and that the commercial dog industry should take action to help prevent the spread of extensively drug-resistant *C jejuni* from pet store puppies to people.

## Key Points

**Question:**

Are pet store puppies a source of extensively drug-resistant *Campylobacter jejuni* infection in the US?

**Findings:**

This survey study identified 168 cases from public health reports of *Campylobacter* infections with an epidemiologic or molecular link to pet store puppies from 2011 to 2020; 97% of patients reported contact with a dog, of whom 88% reported contact with a pet store puppy. Isolates were resistant to 7 antibiotic classes, including all recommended treatment agents.

**Meaning:**

Extensively drug-resistant *C jejuni* strains have emerged as a cause of illness among pet store customers, employees, and visitors; infections caused by these strains cannot be treated with commonly recommended oral antibiotics.

## Teaser

This survey study investigates the epidemiologic, laboratory, and traceback findings associated with persistent, extensively drug-resistant strains of *Campylobacter jejuni* associated with US pet store puppies.

## Introduction

In the US, *Campylobacter* is the most common bacterial cause of diarrhea, with an estimated 1.5 million illnesses and an estimated 450 000 antibiotic-resistant infections each year.^1,2^ The proportion of resistant *Campylobacter* infections has doubled during the last 20 years.^3^ Approximately 30% have decreased susceptibility to fluoroquinolones (eg, ciprofloxacin) or macrolides (eg, azithromycin), agents used to treat severe infections.^1^ Resistant bacterial infections can require longer hospital stays, more medical visits, and more costly treatments with more toxic effects than susceptible infections.^1^

More than 90% of human *Campylobacter* infections are caused by *Campylobacter jejuni*. Major symptoms include diarrhea (often bloody), fever, and abdominal cramps. Most recover within 1 week. Antibiotics are recommended for persons severely ill or at risk for severe disease, including those 65 years or older, infants, pregnant individuals, and immunosuppressed persons.^4^ Macrolides and fluoroquinolones are the recommended antibiotic classes.^4^

In August 2017, the Florida Department of Health received reports of 6 patients diagnosed with *C jejuni* infections who reported contact with puppies sold by a national pet store chain based in Ohio. Samples from puppies yielded isolates highly related by whole-genome sequencing (WGS) to an isolate from a patient in Ohio who had recently purchased a puppy from the same pet store chain. In response, the Centers for Disease Control and Prevention (CDC), along with federal and state partners, initiated a national outbreak investigation of *C jejuni* infections linked to pet store puppies.^5^

From August 1, 2017, to February 29, 2020, we conducted 2 investigations and enhanced surveillance of illnesses linked to pet store puppies. In this report, we summarize the epidemiologic, laboratory, and traceback findings to characterize these persistent, extensively drug-resistant strains.

## Methods

### Data Collection, Case Definitions, and Investigations

Since 1996, the CDC has conducted *Campylobacter* surveillance predominantly through 10 sentinel sites of the Foodborne Diseases Active Surveillance Network (FoodNet), which encompasses 15% of the US population; a subset of isolates from FoodNet sites is submitted to the National Antimicrobial Resistance Monitoring System (NARMS) laboratory for antibiotic susceptibility testing.^3^ All state laboratories submit isolate information to PulseNet, the national molecular subtyping network for foodborne disease surveillance at the CDC.^6^ PulseNet introduced WGS as a *Campylobacter* subtyping method in 2015 and replaced pulsed-field gel electrophoresis as the primary method for *C jejuni* subtyping by October 2018. Some state public health laboratories performed WGS on older isolates. Additional background on *Campylobacter* surveillance in the US is provided in eFigure 1 in the Supplement. To understand the epidemiologic mechanisms of extensively drug-resistant strains, we conducted case finding and investigation in 4 periods. We merged information on culture-confirmed cases collected during 2 investigations, a period of enhanced surveillance, and retrospective case finding. Patients gave verbal informed consent to be interviewed. Laboratory analysis of *C jejuni* specimens was considered to be part of public health surveillance, and consent was not required. Race and ethnicity were assessed by patient self-report. Data were considered coded (not deidentified) because state health departments maintain records that include patient identifiers such as name or address. The CDC epidemiologists did not request and did not receive patient identifiers as a result of this work. Both investigations and the enhanced surveillance protocol were reviewed by the CDC and were consistent with applicable federal law and CDC policy.

### Investigation 1

For this report, we defined a case as culture-confirmed *C jejuni* infection in a patient with (1) an epidemiologic association with a pet store puppy (defined as contact with a pet store puppy before or after purchase, including contact resulting from pet store employment or during pet store visitation) or (2) an isolate highly related by core genome multilocus sequence typing (cgMLST)^7^ to an isolate from a patient with an epidemiologic association. State and local public health officials interviewed patients with cases from January 1, 2016, to February 12, 2018, using a focused questionnaire that included demographic characteristics (age, sex, race, ethnicity, and state of residence), outcomes (hospitalization or death), and exposures 7 days before illness began (contact with a dog or puppy, type of exposure, pet store, or breeder affiliation). During 3 weeks in October 2017, public health officials collected fecal specimens from puppies at implicated pet stores in Kentucky, Ohio, Pennsylvania, and Wisconsin and transported them in Cary Blair media to state laboratories for culture and WGS. Investigators collected information about breeders, distributors, and transporters for all sampled pet store puppies. We conducted traceback of puppies that (1) had a sample with a *C jejuni* isolate highly related to investigation strains by cgMLST or (2) had an epidemiologic association with an infected patient with *Campylobacter* infection (including patients diagnosed by polymerase chain reaction only). We obtained information from state-led investigations.

### Enhanced Surveillance

After investigation 1, we conducted enhanced surveillance from February 13 to December 31, 2018, for illness caused by the same cgMLST-defined strains. State public health laboratories transmitted WGS data through PulseNet. Then, CDC investigators used cgMLST- and a ResFinder, version 3.0 (Center for Genomic Epidemiology)–based workflow to identify related isolates, including those from investigation 1 for which sequencing was performed later. State and local health departments collected information from patients about exposures using a shortened version of the investigation 1 questionnaire.

### Investigation 2

In response to identification of ongoing cases, we conducted an investigation in from January 1, 2019, to February 29, 2020. We defined a case as a culture-confirmed *C jejuni* infection with a strain highly related by cgMLST to an isolate from a patient in investigation 1 or to an isolate linked to a pet store puppy. Health officials interviewed patients with the questionnaire used for enhanced surveillance.

### Retrospective Case Finding

Beginning in 2019, we regularly screened all *Campylobacter* sequences uploaded to PulseNet for genetic relatedness to study isolates to identify isolates from cases that occurred before January 2016 and were sequenced later. We obtained information from interviews health officials had conducted with these patients.

### Isolate Sequencing and Antibiotic Susceptibility Testing

Whole-genome sequencing was performed using PulseNet guidelines. We compared sequences uploaded to PulseNet by cgMLST and estimated isolate relatedness by number of allele differences.^7^ We generated de novo assemblies using Shovill software, version 1.0.9,^8^ analyzed them for resistance determinants using the ResFinder database (90% identity and 50% cutoff), and screened for *gyrA* mutations using the PointFinder scheme for *Campylobacter* species implemented in Staramr software, version 0.4.0.^9^ We identified mutations in the 23S ribosomal RNA region using ARIBA (Sanger Pathogens), version 2.12.0.^10^ Sequence accession numbers are provided in eTable in the Supplement.

All study isolates submitted to the CDC underwent testing for susceptibility to 9 agents from 7 antibiotic classes using a standard broth microdilution assay, CAMPY panel from Sensititre (Thermo Fisher Scientific) per the manufacturer’s directions.^11,12^ Antibiotic classes included aminoglycosides (gentamicin), ketolides (telithromycin), lincosamides (clindamycin), macrolides (azithromycin and erythromycin), quinolones (ciprofloxacin and nalidixic acid), phenicols (florfenicol), and tetracyclines (tetracycline).

We classified isolates as susceptible or resistant using the European Committee on Antimicrobial Susceptibility Testing epidemiological cutoff values or clinical break points.^13,14,15^ We categorized an isolate as resistant if it had a mean inhibitory concentration above the clinical break point (ciprofloxacin and erythromycin)^13^ or epidemiologic cutoff value (azithromycin, clarithromycin, florfenicol, gentamicin, nalidixic acid, telithromycin, and tetracycline).^14,15^ For isolates not tested phenotypically, we predicted resistance based on the presence of known resistance determinants in the genome.^16^ We defined extensively drug resistant as resistant to macrolides and fluoroquinolones (the antibiotic classes recommended for treatment of *Campylobacter*)^4^ and 3 or more additional antibiotic classes.^7,17^

### Statistical Analysis

We described the epidemiologic characteristics of patients and compared proportions with given characteristics from the 2 investigations using χ^2^ analysis or the Fisher exact test for a cell size of 5 or less (a 2-sided *P* ≤ .05 was considered to be statistically significant). All calculations were performed using SAS statistical software, version 9.4 (SAS Institute Inc) or Epi Info, version 7.2.3.1 (CDC). The phylogenetic tree was annotated using Interactive Tree of Life, version 5 (BioByte Solutions).^18^

## Results

### Epidemiologic Analysis

A total of 168 patients (median [interquartile range] age, 37 [19.5-51.0] years; 105 of 163 female [64%]) with an epidemiologic or molecular association with pet store puppies were identified from February 2, 2011, to February 20, 2020 (Table 1). Thirty-one of 126 (25%) with known hospitalization status were hospitalized; none died.

**Table 1.** Characteristics of Patients With Culture-Confirmed *Campylobacter jejuni* Infections Associated With Contact With Pet Store Puppies in the United States, 2011-2020^a^

Characteristic^b^	Total (February 1, 2011, to February 29, 2020) (N = 168)^c^	Investigation 1 (January 1, 2016, to February 29, 2018) (n = 48)	Investigation 2 (January 1, 2019, to February 29, 2020) (n = 42)	*P* value for investigation 1 vs investigation 2
Age median (IQR), y	37 (19.5-51.0)	36.5 (17.5-49.0)	36 (18.0-51.5)	NA
Age group, y				
<5	13/164 (8)	2/48 (4)	6/40 (15)	.13
5-17	20/164 (12)	10/48 (21)	4/40 (10)	.24
18-64	121/164 (74)	34/48 (71)	26/40 (65)	.65
≥65	10/164 (6)	2/48 (4)	4/40 (10)	.41
Sex				
Female	105/163 (64)	33/48 (69)	23/40 (58)	.38
Male	58/163 (36)	15/48 (31)	17/40 (42)	
Race or ethnicity				
Black or African American	8/92 (9)	4/26 (15)	1/20 (5)	.64
Hispanic	6/89 (6)	2/25 (8)	3/20 (15)	.88
White	84/92 (91)	22/26 (85)	18/20 (95)	.37
Geographic region^d^				
Northeast	44/168 (26)	13/48 (27)	3/42 (7)	.02
Midwest	67/168 (40)	21/48 (44)	21/42 (50)	.67
South	29/168 (17)	7/48 (15)	8/42 (19)	.59
West	28/168 (17)	7/48 (15)	10/42 (24)	.40
Hospitalization	31/126 (25)	14/44 (32)	6/32 (19)	.31
LOS, median (range), d^e^	3 (1-31)	3 (2-31)	NA	NA
Death	0/135	0/48	0/37	NA
Exposures				
Any dog or puppy contact	117/121 (97)	40/42 (95)	30/31 (97)	.72
Any pet store puppy contact	69/78 (88)	38/41 (93)	20/24 (83)	.45
Store customer	34/60 (57)	16/29 (55)	12/20 (60)	.97
Store employee	20/60 (33)	10/29(34)	6/20 (30)	.98
Store visitor	3/60 (5)	3/29 (10)	0/20	NA
Other	3/60 (5)	0/29	2/20 (10)	NA
Company affiliation				
Common pet store chain	38/63 (60)	21/31 (68)	12/20 (60)	.79
Other company	22/63 (35)	9/31 (29)	8/20 (40)	.61
No company affiliation	3/63 (5)	1/31 (3)	0/20	NA

Abbreviations: IQR, interquartile range; LOS, length of stay; NA, not applicable.

^a^ Data are presented as number/total number (percentage) of patients unless otherwise indicated.

^b^ Proportions of patients from investigation 1 and investigation 2 were compared by χ^2^ analysis (Fisher exact test was used for calculations with a cell value of ≤5).

^c^ Total includes 48 patients from investigation 1, 41 patients from investigation 2, 45 patients from enhanced surveillance (February 1 to December 31, 2018), and 31 patients from retrospective case finding (before 2016).

^d^ Northeast includes Connecticut, Maryland, Massachusetts, New Hampshire, and New York; Midwest includes Illinois, Iowa, Kansas, Michigan, Minnesota, Missouri, Nebraska, Ohio, and Wisconsin; South includes Florida, Georgia, Kentucky, South Carolina, and Tennessee; and West includes Nevada, Oregon, Utah, and Wyoming.

^e^ No information on length of stay was available for patients from investigation 2.

**Figure 1.**
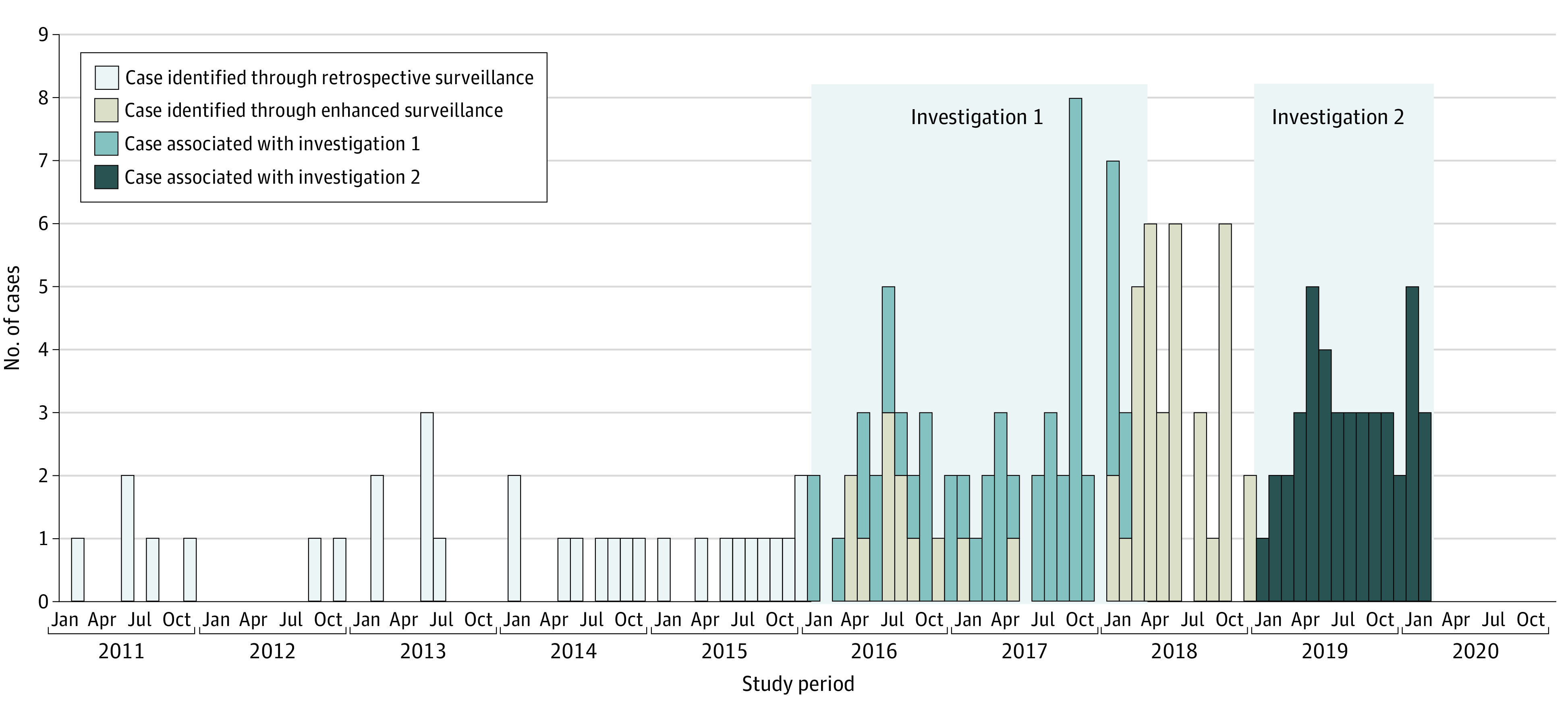
Cases of Culture-Confirmed *Campylobacter jejuni* Infection Linked to Contact With Pet Store Puppies, by Month, US, 2011-2020 Shaded areas show the periods of investigation 1 and investigation 2. Fourteen cases that occurred during the investigation 1 period were identified later because of delayed sequencing of the isolates.

Forty-eight cases were included in investigation 1; 14 more occurred during the same period but were identified during enhanced surveillance (Figure 1). We identified 42 cases during investigation 2, 33 between these investigations as a result of enhanced surveillance, and 31 with patients’ illnesses before 2016. A total of 137 patients had illness onsets from January 8, 2016, to February 20, 2020. More patients were from northeastern states in investigation 1 than in investigation 2 (27% vs 7.1%, *P* = .02); otherwise, study periods did not differ significantly in patient demographic characteristics, outcomes, or exposures.

**Figure 2.**
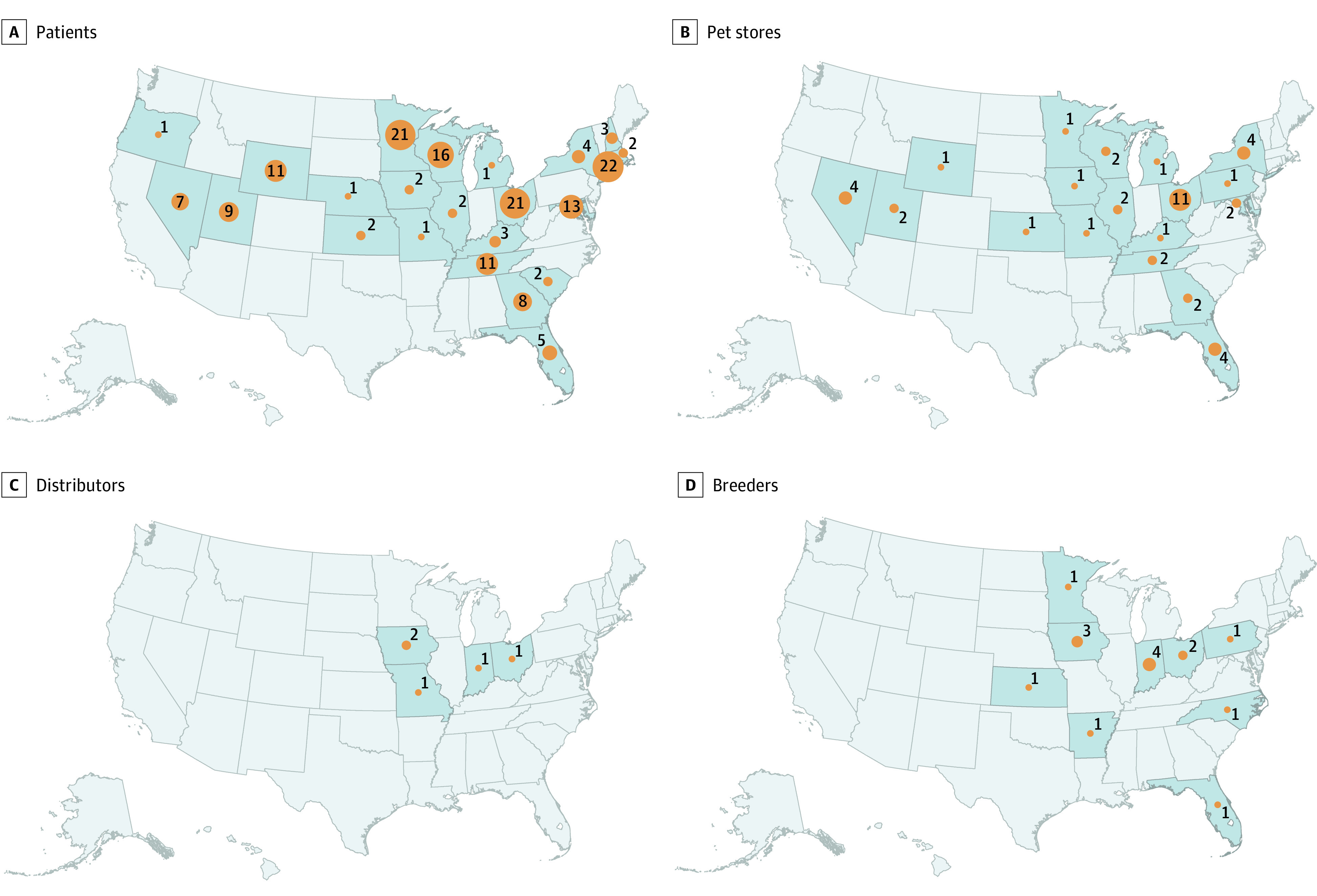
Maps by State Showing Patients With Culture-Confirmed *Campylobacter jejuni* Infection, US, 2011-2020 A, Infections linked to contact with pet store puppies (n = 168). B, Location of affiliated pet stores, when known (n = 43). C, Location of affiliated distributors, when known (n = 5). D, Location of affiliated breeders, when known (n = 15).

Overall, 117 of 121 patients (97%) reported contact with a dog in the week before symptoms began (Table 1). Among patients with additional information, 69 of 78 (88%) reported contact with a puppy from a pet store; 34 of 60 (57%) were customers, 20 of 60 (33%) were pet store employees, and 3 of 60 (5%) were pet store visitors. Among 63 patients with information, 38 (60%) had exposure to puppies from pet stores in 10 states affiliated with a common pet store chain (Figure 2), 22 (35%) had exposure to 16 stores of unrelated companies in 11 states, and 3 (5%) reported contact with puppies purchased directly from breeders in Florida, Kansas, and North Carolina unaffiliated with pet stores. No patients reported contact with puppies from a shelter or rescue organization.

### Pet Store Investigations and Traceback (Investigation 1)

Investigators visited 33 pet stores and collected fecal samples from 211 puppies, including 5 whose specimens yielded a *C jejuni* isolate highly related to investigation strains by cgMLST. State investigators traced 8 additional puppies that were epidemiologically linked to patients. Breeder, distributor, and transportation company information was available for these 13 puppies; each was a different breed from a distinct breeding operation (Figure 2; eFigure 2 in the Supplement). No single breeder, distributor, or transporter was the sole source of infected puppies.

### Isolate Association and Antibiotic Susceptibility

**Figure 3.**
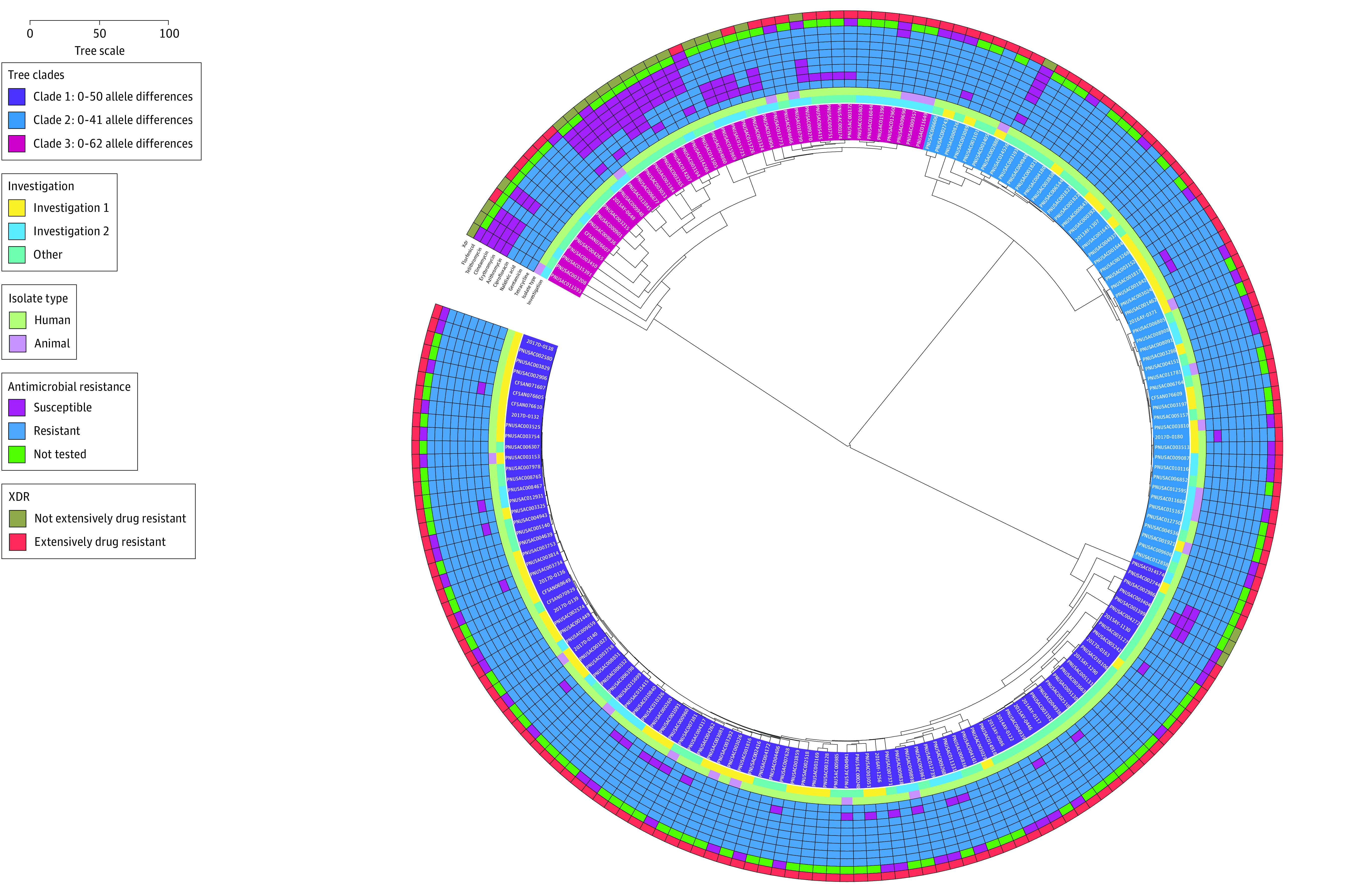
Isolate Relatedness, Investigation Association, and Antibiotic Resistance Patterns of Isolates From Human Patients and Dogs Linked to Pet Stores, US, 2011–2020 Isolate relatedness was assessed using core genome multilocus sequence typing; the figure was generated using Interactive Tree of Life software, version 5 (BioByte Solutions).^18^ Antibiotic resistance determination was based on antibiotic susceptibility testing results when available (n = 72) and otherwise on the presence of known resistance determinants in the bacterial genome for all agents except florfenicol. Isolate identifications are shaded according to the clade to which they belong. Other shaded rings correspond to the investigation type (innermost ring); isolate type; susceptibility status for tetracycline, gentamicin, nalidixic acid, ciprofloxacin, azithromycin, erythromycin, clindamycin, telithromycin, and florfenicol; and extensively drug-resistant (XDR) status (outermost ring). The number of allele differences between isolates is proportionate to the combined distance to the nearest common node; the distance corresponding to 100 allele differences is shown. An interactive version of this figure is available at https://itol.embl.de/shared/2DT03vJtQjoQN.

All human (n = 168) and dog isolates (n = 23, including 5 from puppies identified through traceback) were clustered by cgMLST into 3 clades with allele ranges of 0 to 50 alleles (n = 97), 0 to 41 alleles (n = 53), and 0 to 62 alleles (n = 41); the clades differed from each other by 125 to 1232 alleles (Figure 3). All contained isolates from both humans and dogs. Isolates from investigation 1 fell into 2 clades; isolates from the other periods were distributed across all 3 clades.

One hundred sixty-eight isolates (88%) were extensively drug resistant (Table 2) compared with only 126 of 9358 NARMS surveillance isolates (1.3%) during 2011 to 2019 (*P* < .001); these isolates were distributed throughout all clades (Figure 3). Resistance was significantly higher than among NARMS surveillance isolates for all antibiotic classes (aminoglycosides: 150 of 191 [79%] vs 113 of 9358 [1%]; ketolides: 174 of 191 [91%] vs 201 of 9358 [2%]; lincosamides: 176 of 191 [92%] vs 617 of 9358 [7%]; macrolides: 176 of 191 [92%] vs 200 of 9358 [2%]; phenicols: 13 of 48 [18%] vs 105 of 9358 [1%]; quinolones: 181 of 191 [95%] vs 2441 of 9358 [26%]; and tetracyclines: 191 of 191 [100%] vs 4404 of 9358 [47%]; *P* < .001 for all comparisons). Resistance determinants for antibiotics tested phenotypically on a subset of isolates included the *gyrA* (T86I) mutation (ciprofloxacin and nalidixic acid), 23S mutation (azithromycin, clindamycin, erythromycin, and telithromycin), *tetO* gene (GenBank M18896) (tetracycline), and *aph(2'')-Ih* gene (GenBank KF652096) (gentamicin).^16^ The cause of florfenicol resistance, seen in 13 of 72 isolates (18%) tested phenotypically, was not determined^20^; therefore, florfenicol resistance could not be predicted by WGS. Resistance genes *ant(3′′)-Ia* (GenBank KF864551), *aph(3′)-III* (GenBank M26832), *bla*OXA-61 (GenBank AY587956), *bla*OXA-193 (GenBank CP013032), *bla*OXA-448 (GenBank KR061497), *bla*OXA-453 (GenBank KR061507), and *bla*OXA-461 (GenBank KR061509), which have been associated with decreased susceptibility to streptomycin, kanamycin, amikacin, and β-lactam antibiotics, were present in some isolates; these agents were not tested phenotypically.^16,21,22^

**Table 2.** Antibiotic Resistance of *Campylobacter jejuni* Isolates Associated With Pet Store Puppies (2011-2020) and Surveillance Isolates From the National Antimicrobial Resistance Monitoring System (2011-2019)^a^

Agent	No. (%) of cases			
	All isolates linked to pet store puppies (February 1, 2011, to February 29, 2020) (N = 191)^b^	Investigation 1 (January 1, 2016, to February 29, 2018) (n = 62)	Investigation 2 (January 1, 2019, to February 29, 2020) (n = 44)	NARMS surveillance (January 1, 2011, to December 31, 2019) (N = 9358)^c^
Antimicrobial class				
Quinolones	181 (95)	62 (100)	39 (89)	2441 (26)
Lincosamides	176 (92)	62 (100)	39 (89)	617 (6.6)
Macrolides	176 (92)	62 (100)	39 (89)	200 (2)
Phenicols^c^	13 (18)	10 (30)	2 (8)	105 (1)
Aminoglycosides	150 (79)	53 (85)	32 (73)	113 (1)
Ketolides	174 (91)	62 (100)	38 (86)	201 (2)
Tetracyclines	191 (100)	62 (100)	44 (100)	4404 (47)
XDR^d^	168 (88)	62 (100)	34 (77)	126 (1)

Abbreviation: NARMS, National Antimicrobial Resistance Monitoring System; XDR, extensively drug resistant.

^a^ Antibiotic resistance was determined based on results of antibiotic susceptibility testing when available; otherwise, resistance was determined by the presence of resistance determinants in bacterial genomes. This table includes only antibiotic classes for which phenotypic antibiotic susceptibility testing was performed.

^b^ Total includes isolates from 48 patients from investigation 1, 41 patients from investigation 2, 45 patients from enhanced surveillance (February 1 to December 31, 2018), 31 patients from retrospective case finding (before 2016), and 22 isolates from dogs. Antibiotic resistance was determined by antibiotic susceptibility testing for 73 isolates.

^c^ The NARMS routine surveillance is based on antibiotic susceptibility testing of a subset of isolates from 10 public health laboratories in the FoodNet sites.^19^ During 2011 to 2019, isolates were selected for testing using a frequency-based sampling approach.^3^ These data are based on results of phenotypic antibiotic susceptibility testing only.

^d^ Defined as resistance to macrolides and fluoroquinolones and 3 or more antibiotic classes.

## Discussion

This survey study found that human extensively drug-resistant *C jejuni* infections were associated with contact with puppies sold through the commercial dog industry. Surveillance data indicate the extensively drug-resistant *C jejuni* strains have been circulating for at least 10 years and continue to cause illness among pet store customers, employees, and others who encounter pet store puppies. The extensively drug-resistant isolates are resistant to all recommended treatment agents.^4,7^

Most sporadic *Campylobacter* illnesses in the US have been associated with the consumption of raw or undercooked poultry, international travel, and animal contact.^23,24,25^ A high proportion of antibiotic-resistant *Campylobacter* infections have been associated with international travel.^26^ However, these extensively drug-resistant strains have been associated with only dogs. More than 1 in 3 US households has a dog,^27^ and dogs, especially puppies, can carry *Campylobacter*.^28,29,30^ Dogs carrying *Campylobacter* are frequently asymptomatic,^29^ underscoring the importance of primary prevention among pet store puppies.

Extensively drug-resistant isolates account for only 1.3% of *C jejuni* surveillance isolates submitted to the NARMS during 2011 to 2019.^31^ Antibiotic treatment of extensively drug-resistant *C jejuni* infection requires intravenous antibiotics, such as carbapenems, which are costly and normally reserved for hospital-associated infections. Failure of traditional antibiotics can lead to complications^1^; several patients had prolonged hospital admission after multiple courses of antibiotics to which their strain was resistant.^32^

These strains were circulating in the US for several years before health officials in Florida connected illnesses to pet store puppies through patient interviews. Challenges with public health surveillance and laboratory testing likely contributed to the delay in detection. Many local and state health departments lack resources to routinely obtain exposure information from ill persons. When *Campylobacter* illness clusters are identified, epidemiologists rarely succeed in identifying a common source.^26,33^ No national or regional system routinely collects exposure data to identify common exposures across states. Moreover, the CDC has estimated that only 1 of 30 *Campylobacter* illnesses is ever identified, mainly because most people who are ill with *Campylobacter* infection do not seek medical care, and many who seek care do not have a stool sample collected.^19^ Many clinical laboratories cannot culture *Campylobacter,* which has special growth requirements.^34^

Culture-independent diagnostic tests, such as polymerase chain reaction–based assays and immunoassays, which were used by 9% of clinical laboratories in FoodNet sites in 2012 and 34% in 2019, do not yield an isolate needed for subtyping (eg, WGS) and susceptibility testing.^35,36^ Some clinical laboratories do not forward isolates to their public health laboratory. During investigation 1, at least 70 additional patients, not included in this analysis, had epidemiologic ties to pet store puppies and positive diagnostic test results not confirmed by culture,^5^ illustrating that many cases were likely missed because no isolate is available for subtyping. Pulsed-field gel electrophoresis, the standard subtyping method used by PulseNet for more than 2 decades for detection of multistate outbreaks caused by *Salmonella*, *Listeria*, and *Escherichia coli*, has not worked as well for *Campylobacter*.^7,33,37^ Whole-genome sequencing could help improve *Campylobacter* surveillance and outbreak detection, but *Campylobacter* isolates have been a lower priority for state public health laboratories with limited sequencing capacity. Limited implementation of WGS for *Campylobacter* affected the timeliness and completeness of case identification across study periods.

To our knowledge, the extensively drug-resistant strains were only found in the commercial dog industry and have not been associated with exposure to dogs from animal shelters, indicating these strains might have a niche in commercial breeding and distribution of pet store puppies. During investigation 1, Montgomery et al^5^ found that 95% of dogs received 1 or more antibiotic courses for prophylaxis or empirical treatment at a breeder, transporter, or pet store. Use of antibiotics and other management practices in the commercial dog industry might have selected for extensively drug-resistant strains and facilitated spread among dogs from 1 or more breeding facilities to many stores. In animal agriculture, factors such as crowding and inadequate husbandry have been associated with spread of illnesses among animals that may require antibiotic treatment, resulting in selection of resistant strains^38,39^; similar conditions could be occurring in the commercial dog industry.

Public health recommendations to reduce illness among dogs, customers, and store employees were provided to a common pet store chain, other pet stores, and the general veterinary community.^40,41^ Despite these recommendations, illnesses have continued to occur. The US Department of Agriculture’s Animal and Plant Inspection Service Animal Care program ensures the humane treatment of animals covered by the Animal Welfare Act. However, no regulatory agency oversees antibiotic use in the commercial dog industry; therefore, adoption of infection prevention and antibiotic stewardship recommendations is left to the discretion of individual companies. The commercial dog industry could implement measures to curb unnecessary antibiotic use and improve hygiene and infection control at all levels from breeding facility to pet store, similar to those taken by the food animal production industry under US Food and Drug Administration guidance.^42,43^ Veterinary school curricula, continuing veterinary education focusing on antibiotic stewardship for veterinarians working with the commercial dog industry, and increased veterinary oversight within the industry may improve prescribing practices. A national surveillance system capable of combining human and companion animal diagnostic data could also improve the detection and investigation of zoonotic illness.

### Limitations

Our study has several limitations. The cases we report likely underestimate the total burden of extensively drug-resistant *C jejuni* infections associated with pet store puppies for the multiple reasons described above. We did not obtain exposure data regarding dog or puppy contact for all cases, especially for those that occurred before or between investigations 1 and 2. Traceback data revealed that puppies were often comingled throughout the distribution chain, making the primary source of infected puppies difficult to identify.

## Conclusions

The results of this survey study suggest that practitioners should ask about puppy exposure (including occupational exposure) when treating patients with *Campylobacter* infection, especially those who do not improve with routine antibiotic treatment. When a polymerase chain reaction–based diagnostic test result is positive, an isolate should be obtained from a reflex stool culture for antibiotic susceptibility testing, public health surveillance, and outbreak detection. The commercial dog industry also needs to take action to help prevent the spread of extensively drug-resistant *C jejuni* from pet store puppies to people, including employees. This study highlights an ongoing problem within the companion animal sector that will require a collaborative solution. These results indicate that public health officials, the commercial dog industry, animal welfare advocates, regulatory officials, physicians, and veterinarians should adopt a One Health approach^44^ to prevent the development and slow the spread of antibiotic resistance.

## Notes

Supplement. **eTable.** Study Isolate Accession Numbers—National Center for Biotechnology and Information **eFigure 1.** Surveillance and Investigation Timeline—United States, 1996-2020 **eFigure 2.** Traceback Schematic Showing Breeder and Distributor Information for 13 Puppies With an Epidemiologic or Laboratory Link to a Patient With Extensively Drug-Resistant *Campylobacter jejuni* Infection During Investigation 1 (January 2016-February 2018)
