# Supplementary material for: Ongoing Outbreak of Extensively Drug-Resistant Campylobacter jejuni Infections Associated With US Pet Store Puppies, 2016-2020
Source: JAMA Netw Open. 2021 Sep 15;4(9):e2125203. doi: 10.1001/jamanetworkopen.2021.25203 (PMC8444031; doi:10.1001/jamanetworkopen.2021.25203)
Supplement: Supplement. — eTable. Study Isolate Accession Numbers—National Center for Biotechnology and Information eFigure 1. Surveillance and Investigation Timeline—United States, 1996-2020 eFigure 2. Traceback Schematic Showing Breeder and Distributor Information for 13 Puppies With an Epidemiologic or Laboratory Link to a Patient With Extensively Drug-Resistant Campylobacter jejuni Infection During Investigation 1 (January 2016-February 2018) [file jamanetwopen-e2125203-s001.pdf]

Supplementary Online Content

Francois Watkins LK, Laughlin ME, Joseph LA, et al. Ongoing outbreak of extensively drug-resistant *Campylobacter jejuni* infections associated with US pet store puppies, 2016-2020. *JAMA Netw Open*. 2021;4(9):e2125203. doi:10.1001/jamanetworkopen.2021.25203

eTable. Study Isolate Accession Numbers—National Center for Biotechnology and Information

eFigure 1. Surveillance and Investigation Timeline—United States, 1996-2020

eFigure 2. Traceback Schematic Showing Breeder and Distributor Information for 13 Puppies With an Epidemiologic or Laboratory Link to a Patient With Extensively Drug-Resistant *Campylobacter jejuni* Infection During Investigation 1 (January 2016-February 2018)

This supplementary material has been provided by the authors to give readers additional information about their work.

eTable. Study isolate accession numbers – National Center for Biotechnology and Information

Strain	Source	Year	BioSample	Run
2013AY-0086	Human	2013	SAMN10834655	SRR8497483
2013AY-1296	Human	2013	SAMN10834665	SRR8497475
2013AY-1307	Human	2013	SAMN10834657	SRR8497481
2014AY-0117	Human	2014	SAMN08098209	SRR6354024
2014AY-0122	Human	2014	SAMN08098208	SRR6354033
2015AY-0446	Human	2015	SAMN08098200	SRR6354030
2015AY-0648	Human	2015	SAMN08098206	SRR6354035
2015AY-1130	Human	2015	SAMN08098202	SRR6354020
2015AY-1290	Human	2015	SAMN08098205	SRR6354025
2016AY-0371	Human	2016	SAMN08098203	SRR6354019
2016AY-1256	Human	2016	SAMN08098204	SRR6354026
2017D-0132	Human	2017	SAMN07615386	SRR6014503
2017d-0136	Animal	2017	SAMN07504897	SRR6171584
2017d-0138	Animal	2017	SAMN07504895	SRR6171584
2017d-0139	Animal	2017	SAMN07504896	SRR6171587
2017d-0140	Animal	2017	SAMN07504898	SRR6171583
2017d-0163	Animal	2017	SAMN07674670	SRR6171585
2017d-0180	Human	2017	SAMN07605519	SRR6202903
Cfsan069649	Animal	2017	SAMN07710399	SRS2544236
Cfsan070929	Animal	2017	SAMN07823471	SRS2631803
Cfsan071607	Animal	2017	SAMN08025804	SRS2693135
Cfsan076605	Animal	2018	SAMN08624512	SRS3025548
Cfsan076607	Animal	2018	SAMN08624510	SRS3025470
Cfsan076609	Animal	2018	SAMN08624509	SRS3025489
Cfsan076610	Animal	2018	SAMN08624507	SRS3025562
PNUSAC000248	Human	2015	SAMN04429779	SRR3145090
PNUSAC000260	Human	2016	SAMN05255136	SRR3710100
PNUSAC000392	Human	2016	SAMN04961829	SRR3492163
PNUSAC000643	Human	2016	SAMN05413992	SRR3932384
PNUSAC000826	Human	2016	SAMN05791954	SRR4280286
PNUSAC000901	Human	2016	SAMN05804544	SRR5017006
PNUSAC000960	Human	2016	SAMN05908962	SRR4427165
PNUSAC001027	Human	2016	SAMN06007277	SRR5060317
PNUSAC001091	Human	2016	SAMN06036245	SRR5039804
PNUSAC001449	Human	2016	SAMN06562215	SRR5337178
PNUSAC001462	Human	2016	SAMN06285341	SRR5230136
PNUSAC001641	Human	2017	SAMN06644938	SRR5382106
PNUSAC001644	Human	2017	SAMN06644941	SRR5382101
PNUSAC001816	Human	2017	SAMN06759833	SRR5456705
PNUSAC001817	Human	2017	SAMN06759834	SRR5456706
PNUSAC001821	Human	2013	SAMN06829855	SRR5483502
PNUSAC001822	Human	2013	SAMN06829856	SRR5483503
PNUSAC001823	Animal	2013	SAMN06829857	SRR5483512
PNUSAC001842	Human	2017	SAMN07135004	SRR5581839
PNUSAC001921	Human	2017	SAMN07124712	SRR5581935
PNUSAC002384	Human	2016	SAMN07419407	SRR5878362
PNUSAC002416	Human	2017	SAMN07629386	SRR6048525
PNUSAC002510	Human	2015	SAMN07490848	SRR5932396
PNUSAC002518	Human	2017	SAMN07510042	SRR5937910

© 2021 Francois Watkins LK et al. *JAMA Network Open.*

Strain	Source	Year	BioSample	Run
PNUSAC002574	Animal	2017	SAMN07504894	SRR5935033
PNUSAC002580	Animal	2017	SAMN07504900	SRR5935048
PNUSAC002743	Human	2017	SAMN07646583	SRR6048555
PNUSAC002744	Human	2017	SAMN07646584	SRR6048556
PNUSAC002880	Human	2017	SAMN07761796	SRR6152533
PNUSAC002906	Human	2017	SAMN07790191	SRR6179762
PNUSAC002986	Human	2011	SAMN07811994	SRR6202926
PNUSAC003024	Human	2016	SAMN07837859	SRR6232441
PNUSAC003026	Human	2016	SAMN07837861	SRR6232434
PNUSAC003029	Human	2017	SAMN07837874	SRR6232448
PNUSAC003033	Human	2017	SAMN07837867	SRR6232462
PNUSAC003051	Human	2016	SAMN07837858	SRR6232430
PNUSAC003081	Human	2017	SAMN07988994	SRR6313161
PNUSAC003089	Human	2016	SAMN07977045	SRR6261379
PNUSAC003101	Human	2011	SAMN07967650	SRR6252249
PNUSAC003152	Human	2017	SAMN08014318	SRR6289219
PNUSAC003153	Human	2017	SAMN08014319	SRR6289213
PNUSAC003169	Human	2017	SAMN08016191	SRR6289218
PNUSAC003172	Animal	2017	SAMN08016801	SRR6289215
PNUSAC003174	Animal	2017	SAMN08016803	SRR6289217
PNUSAC003192	Human	2014	SAMN08050093	SRR6315880
PNUSAC003193	Human	2014	SAMN08050094	SRR6315883
PNUSAC003194	Human	2015	SAMN08050095	SRR6315887
PNUSAC003197	Human	2017	SAMN08050098	SRR6315881
PNUSAC003208	Animal	2017	SAMN08042328	SRR6308365
PNUSAC003215	Human	2015	SAMN08045651	SRR6313024
PNUSAC003228	Human	2017	SAMN08135041	SRR6353935
PNUSAC003265	Human	2014	SAMN08057557	SRR6322616
PNUSAC003268	Human	2017	SAMN08113727	SRR6337383
PNUSAC003286	Human	2017	SAMN08115708	SRR6337362
PNUSAC003292	Human	2017	SAMN08227096	SRR6420461
PNUSAC003324	Animal	2017	SAMN08114841	SRR6337349
PNUSAC003325	Human	2017	SAMN08114840	SRR6337345
PNUSAC003399	Human	2011	SAMN08235481	SRR6420455
PNUSAC003404	Human	2011	SAMN08235483	SRR6420452
PNUSAC003405	Human	2011	SAMN08235484	SRR6420457
PNUSAC003450	Human	2017	SAMN08239965	SRR6424104
PNUSAC003513	Animal	2017	SAMN08240012	SRR6424098
PNUSAC003525	Human	2017	SAMN08285080	SRR6439804
PNUSAC003663	Human	2012	SAMN08391248	SRR6993873
PNUSAC003734	Human	2018	SAMN08513612	SRR6703680
PNUSAC003751	Human	2018	SAMN08517333	SRR6706106
PNUSAC003753	Human	2018	SAMN08556545	SRR6741489
PNUSAC003754	Animal	2018	SAMN08556540	SRR6741488
PNUSAC003758	Human	2017	SAMN08541473	SRR6728403
PNUSAC003810	Human	2018	SAMN08615568	SRR6785781
PNUSAC003814	Human	2018	SAMN08729633	SRR6856517
PNUSAC003829	Human	2018	SAMN08633868	SRR6801479
PNUSAC003858	Human	2018	SAMN08849554	SRR6929981
PNUSAC003859	Human	2018	SAMN08849550	SRR6929982
PNUSAC004151	Human	2018	SAMN08935219	SRR7007280
PNUSAC004161	Human	2018	SAMN08921938	SRR6993877

© 2021 Francois Watkins LK et al. *JAMA Network Open.*

Strain	Source	Year	BioSample	Run
PNUSAC004172	Human	2018	SAMN08976119	SRR7059394
PNUSAC004180	Human	2018	SAMN08935226	SRR7007273
PNUSAC004262	Human	2018	SAMN09002344	SRR7083212
PNUSAC004265	Human	2018	SAMN09008081	SRR7089338
PNUSAC004272	Human	2018	SAMN09008083	SRR7089365
PNUSAC004317	Human	2018	SAMN09070521	SRR7118963
PNUSAC004406	Human	2018	SAMN09210563	SRR7166319
PNUSAC004536	Human	2018	SAMN09223455	SRR7182410
PNUSAC004639	Human	2018	SAMN09254485	SRR7214227
PNUSAC004666	Human	2018	SAMN09380202	SRR7276867
PNUSAC004860	Human	2018	SAMN09429079	SRR7346454
PNUSAC004937	Human	2014	SAMN09504230	SRR7443911
PNUSAC004938	Human	2014	SAMN09504228	SRR7443905
PNUSAC004939	Human	2014	SAMN09435849	SRR7352584
PNUSAC004940	Human	2015	SAMN09435850	SRR7352588
PNUSAC004941	Human	2015	SAMN09504229	SRR7443915
PNUSAC004943	Human	2017	SAMN09504228	SRR7443905
PNUSAC005127	Human	2018	SAMN09671544	SRR7532444
PNUSAC005130	Human	2012	SAMN09653823	SRR7517840
PNUSAC005131	Human	2013	SAMN09545624	SRR7468590
PNUSAC005140	Human	2018	SAMN09631646	SRR7499439
PNUSAC005157	Human	2018	SAMN09604156	SRR7472643
PNUSAC005242	Human	2018	SAMN09636742	SRR7504911
PNUSAC005415	Human	2018	SAMN09727298	SRR7611019
PNUSAC005961	Human	2018	SAMN09941072	SRR7773444
PNUSAC005985	Human	2018	SAMN09984769	SRR7795433
PNUSAC006307	Human	2018	SAMN10163374	SRR7949763
PNUSAC006398	Human	2018	SAMN10180455	SRR7968377
PNUSAC006544	Human	2018	SAMN10266281	SRR8083469
PNUSAC006552	Human	2018	SAMN10260430	SRR8079775
PNUSAC006606	Human	2018	SAMN10287185	SRR8103790
PNUSAC006677	Human	2018	SAMN10438495	SRR8203994
PNUSAC006764	Human	2018	SAMN10397952	SRR8177998
PNUSAC006805	Human	2018	SAMN10397946	SRR8178042
PNUSAC006816	Human	2018	SAMN10422374	SRR8187086
PNUSAC006852	Human	2018	SAMN10589348	SRR8306535
PNUSAC007371	Human	2018	SAMN10724952	SRR8434313
PNUSAC007381	Human	2018	SAMN10724943	SRR8434305
PNUSAC007628	Human	2018	SAMN10714797	SRR8418787
PNUSAC007978	Human	2017	SAMN10867884	SRR8525870
PNUSAC008091	Human	2019	SAMN11022492	SRR8632918
PNUSAC008467	Human	2019	SAMN11109455	SRR8715354
PNUSAC008765	Human	2018	SAMN16078535	SRR12606552
PNUSAC008851	Human	2019	SAMN11250476	SRR8782863
PNUSAC008908	Human	2019	SAMN11439804	SRR8914616
PNUSAC009087	Human	2019	SAMN11490887	SRR8950402
PNUSAC009268	Human	2019	SAMN14396413	SRR11342509
PNUSAC009270	Human	2019	SAMN11582981	SRR9019622
PNUSAC009357	Human	2019	SAMN14396415	SRR11342510
PNUSAC009606	Human	2019	SAMN14390643	SRR11321380
PNUSAC009659	Human	2019	SAMN14608373	SRR11557474
PNUSAC009690	Human	2019	SAMN14396414	SRR11342520

© 2021 Francois Watkins LK et al. *JAMA Network Open.*

Strain	Source	Year	BioSample	Run
PNUSAC009836	Human	2019	SAMN12108601	SRR9335747
PNUSAC009838	Human	2019	SAMN12108597	SRR9335745
PNUSAC009898	Human	2019	SAMN12307459	SRR9705411
PNUSAC009948	Human	2019	SAMN12127688	SRR9593495
PNUSAC010116	Human	2019	SAMN12421851	SRR9879560
PNUSAC010326	Human	2019	SAMN12528564	SRR9921270
PNUSAC010799	Human	2019	SAMN12504884	SRR9901717
PNUSAC010840	Human	2019	SAMN12518212	SRR9915954
PNUSAC011331	Human	2019	SAMN12642726	SRR10024862
PNUSAC011593	Human	2019	SAMN12736016	SRR10103059
PNUSAC011649	Human	2019	SAMN12745436	SRR10116195
PNUSAC011680	Human	2019	SAMN12766991	SRR10124058
PNUSAC011781	Human	2019	SAMN12830807	SRR10174197
PNUSAC012595	Human	2019	SAMN13032462	SRR10281749
PNUSAC012739	Human	2019	SAMN13058937	SRR10311572
PNUSAC012750	Human	2019	SAMN13058811	SRR10310359
PNUSAC012858	Human	2017	SAMN13087375	SRR10325876
PNUSAC012931	Human	2019	SAMN13116031	SRR10348600
PNUSAC012960	Human	2019	SAMN13116628	SRR10348703
PNUSAC013773	Human	2019	SAMN13392908	SRR10536684
PNUSAC013845	Human	2019	SAMN13473218	SRR10579508
PNUSAC014266	Human	2019	SAMN13656684	SRR10747831
PNUSAC014287	Human	2016	SAMN13634673	SRR10738420
PNUSAC014505	Human	2016	SAMN13977250	SRR11007825
PNUSAC014526	Human	2016	SAMN14546233	SRR11492192
PNUSAC014574	Human	2019	SAMN13699046	SRR10803555
PNUSAC014904	Human	2019	SAMN14049758	SRR11032272
PNUSAC015167	Human	2020	SAMN13897151	SRR10919697
PNUSAC015390	Human	2020	SAMN13975226	SRR11004666
PNUSAC015391	Human	2020	SAMN13975217	SRR11004664
PNUSAC015415	Human	2020	SAMN13977299	SRR11007929
PNUSAC015699	Human	2020	SAMN14088458	SRR11073224
PNUSAC015725	Animal	2020	SAMN14124791	SRR11100741
PNUSAC015726	Human	2020	SAMN14124790	SRR11100742
PNUSAC015802	Animal	2020	SAMN14169401	SRR11151282
PNUSAC015969	Human	2020	SAMN14267089	SRR11218506
PNUSAC016044	Human	2020	SAMN14332977	SRR11268064
PNUSAC016106	Human	2016	SAMN14345131	SRR11280624

© 2021 Francois Watkins LK et al. *JAMA Network Open.*

eFigure 1. Surveillance and investigation timeline—United States, 1996–2020

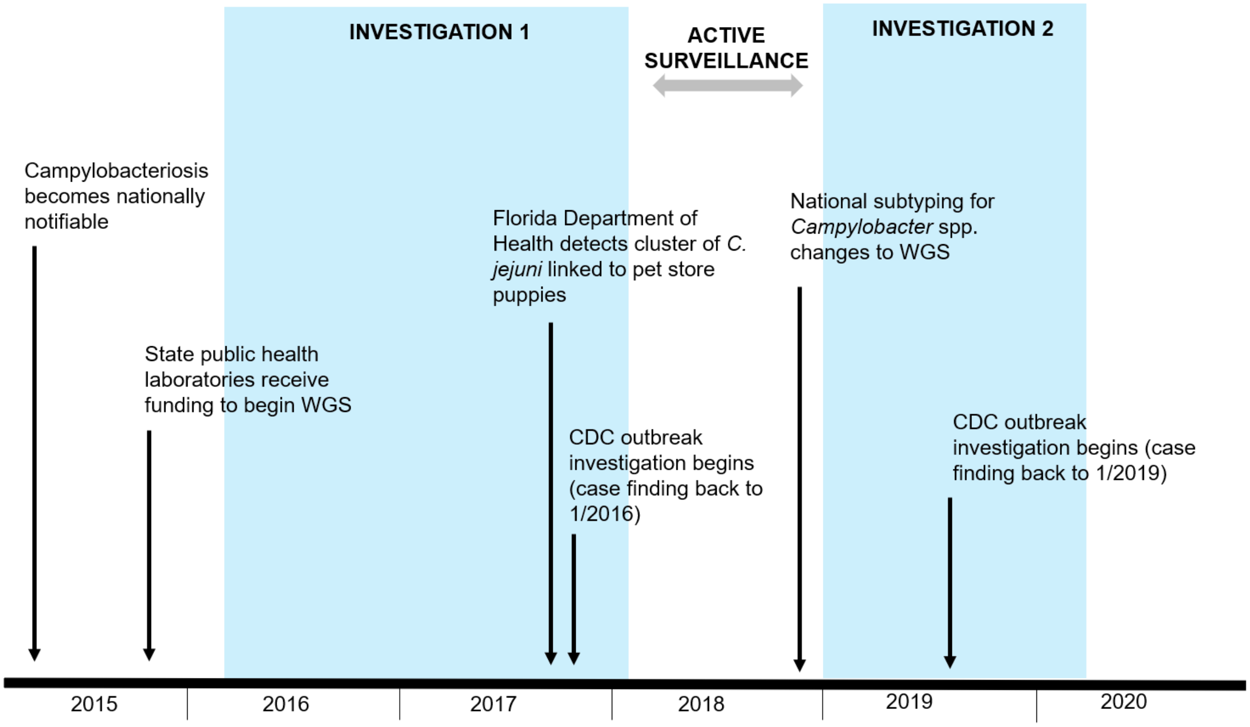

NARMS = National Antimicrobial Resistance Monitoring System; WGS = whole genome sequencing. Highlighted regions show the time periods of two multistate investigations for *Campylobacter jejuni* infections linked to pet store puppies.

Information about surveillance practices can provide a framework for understanding the public health response to extensively drug-resistant *Campylobacter jejuni* infections linked to pet store puppies.

Before 2015, national surveillance for campylobacteriosis was conducted through three primary systems established in 1996: PulseNet, FoodNet, and NARMS. PulseNet (http://www.cdc.gov/pulsenet) is the national laboratory network responsible for detecting outbreaks by linking cases of enteric bacteria disease based on subtyping information submitted from state public health laboratories. Initially, pulsed-field gel electrophoresis (PFGE) was the primary subtyping method used by PulseNet. Few states performed PFGE on *Campylobacter* isolates before the early 2000s when *Campylobacter*-specific protocols were developed; practices varied by state, with many performing PFGE on only a subset of isolates and some not at all. FoodNet (Foodborne Diseases Active Surveillance Network (http://www.cdc.gov/foodnet), is a laboratory-based surveillance system comprising sites in 10 states that cover approximately 15% of the U.S. population. FoodNet collects demographics, epidemiologic information, and outcome data about patients with *Campylobacter* infection. Public health laboratories affiliated with FoodNet sites submit of *Campylobacter* isolates to CDC's NARMS laboratory (http://www.cdc.gov/narms) for antibiotic susceptibility testing using a frequency-based sampling scheme.

In 2015, campylobacteriosis became nationally notifiable, a change that prompted some health departments to improve their surveillance programs. The same year, state public health laboratories received funding to begin implementing WGS of enteric pathogens, and by October 2018, core genome multilocus sequence typing (cgMLST) based on WGS replaced PFGE as PulseNet's primary subtyping modality for *Campylobacter* spp. Some state laboratories also sequenced older isolates, making it possible to identify related strains from earlier times.

However, challenges to *Campylobacter* surveillance have persisted. The organism can be difficult to culture and requires trained laboratory personnel. The increased availability of culture-independent PCR-based tests during the 2010s has had a dramatic impact on diagnostic testing. Many clinical and public health laboratories are not equipped to perform reflex culture of positive PCR tests, so many *Campylobacter* diagnoses do not result in an isolate (required for assessing relatedness to an outbreak strain or susceptibility testing). Not all states require isolates be submitted to state public health laboratories, and reporting delays often result in isolates being discarded by clinical laboratories before public health officials are aware of the case. Furthermore, although WGS holds great promise for improving outbreak detection, the process is costly; many public health laboratories cannot sequence all isolates due to resource constraints.

© 2021 Francois Watkins LK et al. *JAMA Network Open.*

eFigure 2. Traceback schematic showing breeder and distributor information for 13 puppies with an epidemiologic or laboratory link to a patient with extensively drug-resistant *Campylobacter jejuni* infection during Investigation 1 (January 2016–February 2018)

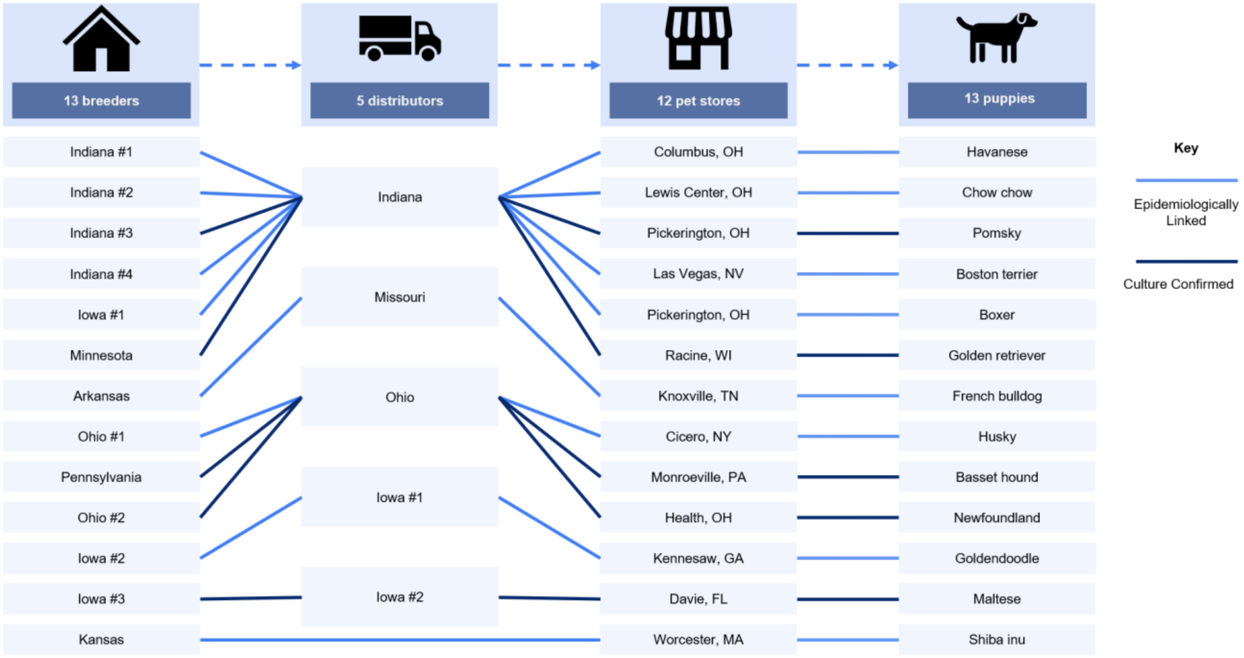

© 2021 Francois Watkins LK et al. *JAMA Network Open.*
